# Supplementary material for: Characterization of the First Secreted Sorting Nexin Identified in the Leishmania Protists
Source: Int J Mol Sci. 2024 Apr 7;25(7):4095. doi: 10.3390/ijms25074095 (PMC11012638; doi:10.3390/ijms25074095)
Supplement: Supplementary file 1 [file ijms-25-04095-s001.zip › LdSNXi sequence.pdf]

A. *Ld*SNXi cDNA Sequence (*L.donovani* strain LG13)

ATGGCCGCCGGCAACTCCATCTCTGTAAAGGTGGATGTGCCGTCCCAGG
TGAAGGGCCAGGGCACTCTGGAGATGTCGTACTACATCTACCCCATCAC
GATGCGCTTGCCTGGCTTTATGAGCGATGCTCGGTTCAACCGGCGGTAC
ACCGACTTTGAGACTCTGCGCGGCCAGCTGTGTGCGACCTACTGGTACT
GCATTGTGCCACCCATTCCAGAGAAGGAGTCGGTGCAGGATAAGCTGGG
AAAGCTGCCACGCATGGTGGCGTCGGCGAAGGAGACGACCGCCTCCGAG
GGCGACCTGCTCGAGTACCGTCGTATCAGCCTGCGGCGCTTCCTGCAGC
GGCTTGCCTACCACCCCATCCTGGGCAAGTCTGACCTTCTACAGAAGTT
CACGAACGATAACGAGTGGCGCCAGTGCACTCGCGACCCGGTGAAGCCG
CCTCGCTTTATCGTGTCGTCGTCGCTGGAGGAGATCGCCAGATCATGGG
CTCCGTCAAGCAGTGCCTCTGGCGCCGGTACTCAAGGTAGCGGTGGCGC
TGCCGGGGGTGGTGGCGTTAGCCAAACCGGAGCGGCGTATCAGTTGGCA
CTGACTCAAGAGCCAGTGGATGAGGCGACGTGGAGGGCGACAAGCGAGT
ACATTGGTGAGCTGGAGTCTAACCTCAAGAGCATGCGCAACCTGCTGGA
GGCACTCGTGGATCGTCACCGGCGCGCCGCCTCCGCCGTGAGCAACTTC
GCCGCCTCTTTTGGGCTGCTTGCGGAAGGCGAAGAGGATGCCGAGCTGC
GCGGCGCCATCGAAGGGGTGCGCGACTGCGGTCGCAAAGTGGCAGATGT
CTACAGCAAACACGCTGACAACGAGTCGACGCGGCTTGTCAGCACGTTA
AGCTTTTACGTGGGTATGTGTGCCGCCGTGCGGGAGACTCTGAGCCACA
TGTTCAGCGCCCGCCAGTATCTGCGCAACTTGCAGAAGAAGGGCCAGGA
ACTGCAGGCGTCGGCGATGCGGGCACAGTCGGCCAACCAGGTGCAGCTG
CAGAGCGAGCTGCACTTCGTGAATGAGCAGCGGGCCCACCTCGAGGAAG
ACCTCATGGGCGCCGAGAAGACCTTCAGCGAGGAATTTATTCTCTTCCA
CGAAAACAAGCAATATGACGCCAAGGATATGCTGAAGAAGTTTGGCATG
CTGGAGCTCAGCTTCTCTGAGTCGATGAAGCAGGAGTGGGACGCCCTGC
GGCCAATGTTGGAGTCCCTTGGCACATAG

B. *Ld*SNXi Amino acid Sequence (*L.donovani* strain LG13)

MAAGNSISVKVDVPSQVKGQGTLEMSYYIYPITMRLPGFMSDARFNRRY
TDFETLRGQLCATYWYCIVPPIPEKESVQDKLGKLPRMVASAKETTASE
GDLLEYRRISLRRFLQRLAYHPILGKSDLLQKFTNDNEWRQCTRDPVKP
PRFIVSSSLEEIARSWAPSSSASGAGTQGSGGAAGGGGVSQTGAAYQLA
LTQEPVDEATWRATSEYIGELESNLKSMRNLLEALVDRHRRAASAVSNF
AASFGLLAEGEEDAELRGAIEGVRDCGRKVADVYSKHADNESTRLVSTL
SFYVGMCAAVRETLSHMFSARQYLRNLQKKGQELQASAMRAQSANQVQL
QSELHFVNEQRAHLEEDLMGAEKTFSEEFILFHENKQYDAKDMLKKFGM
LELSFSESMKQEWDALRPMLESLGT

CLUSTAL O(1.2.4) multiple sequence alignment

DNA Sequence

```
LdSNXi            ATGGCCGCCGGCAACTCCATCTCTGTAAAGGTGGATGTGCCGTCCCAGGTGAAGGGCCAG     60
LDBPK_352470.1    ATGGCCGCCGGCAACTCCATCTCTGTAAAGGTGGATGTGCCGTCCCAGGTGAAGGGCCAG     60
                  ************************************************************

LdSNXi            GGCACTCTGGAGATGTCGTACTACATCTACCCCATCACGATGCGCTTGCCTGGCTTTATG     120
LDBPK_352470.1    GGCACTCTGGAGATGTCGTACTACATCTACCCCATCACGATGCGCTTGCCTGGCTTTATG     120
                  ************************************************************

LdSNXi            AGCGATGCTCGGTTCAACCGGCGGTACACCGACTTTGAGACTCTGCGCGGCCAGCTGTGT     180
LDBPK_352470.1    AGCGATGCTCGGTTCAACCGGCGGTACACCGACTTTGAGACTCTGCGCGGCCAGCTGTGT     180
                  ************************************************************

LdSNXi            GCGACCTACTGGTACTGCATTGTGCCACCCATTCCAGAGAAGGAGTCGGTGCAGGATAAG     240
LDBPK_352470.1    GCGACCTACTGGTACTGCATTGTGCCACCCATTCCAGAGAAGGAGTCGGTGCAGGATAAG     240
                  ************************************************************

LdSNXi            CTGGGAAAGCTGCCACGCATGGTGGCGTCGGCGAAGGAGACGACCGCCTCCGAGGGCGAC     300
LDBPK_352470.1    CTGGGAAAGCTGCCACGCATGGTGGCGTCGGCGAAGGAGACGACCGCCTCCGAGGGCGAC     300
                  ************************************************************

LdSNXi            CTGCTCGAGTACCGTCGTATCAGCCTGCGGCGCTTCCTGCAGCGGCTTGCCTACCACCCC     360
LDBPK_352470.1    CTGCTCGAGTACCGTCGTATCAGCCTGCGGCGCTTCCTGCAGCGGCTTGCCTACCACCCC     360
                  ************************************************************

LdSNXi            ATCCTGGGCAAGTCTGACCTTCTACAGAAGTTCACGAACGATAACGAGTGGCGCCAGTGC     420
LDBPK_352470.1    ATCCTGGGCAAGTCTGACCTTCTACAGAAGTTCACGAACGATAACGAGTGGCGCCAGTGC     420
                  ************************************************************

LdSNXi            ACTCGCGACCCGGTGAAGCCGCCTCGCTTTATCGTGTCGTCGTCGCTGGAGGAGATCGCC     480
LDBPK_352470.1    ACTCGCGACCCGGTGAAGCCGCCTCGCTTTATCGTGTCGTCGTCGCTGGAGGAGATCGCC     480
                  ************************************************************

LdSNXi            AGATCATGGGCTCCGTCAAGCAGTGCCTCTGGCGCCGGTACTCAAGGTAGCGGTGGCGCT     540
LDBPK_352470.1    AGATCATGGGCTCCGTCAAGCAGTGCCTCTGGCGCCGGTACTCAAGGTAGCGGTGGCGCT     540
                  ************************************************************

LdSNXi            GCCGGGGGTGGTGGCGTTAGCCAAACCGGAGCGGCGTATCAGTTGGCACTGACTCAAGAG     600
LDBPK_352470.1    GCCGGGGGTGGTGGCGTTAGCCAAACCGGAGCGGCGTATCAGTTGGCACTGACTCAAGAG     600
                  ************************************************************

LdSNXi            CCAGTGGATGAGGCGACGTGGAGGGCGACAAGCGAGTACATTGGTGAGCTGGAGTCTAAC     660
LDBPK_352470.1    CCAGTGGATGAGGCGACGTGGAGGGCGACAAGCGAGTACATTGGTGAGCTGGAGTCTAAC     660
                  ************************************************************

LdSNXi            CTCAAGAGCATGCGCAACCTGCTGGAGGCACTCGTGGATCGTCACCGGCGCGCCGCCTCC     720
LDBPK_352470.1    CTCAAGAGCATGCGCAACCTGCTGGAGGCACTCGTGGATCGTCACCGGCGCGCCGCCTCC     720
                  ************************************************************

LdSNXi            GCCGTGAGCAACTTCGCCGCCTCTTTTGGGCTGCTTGCGGAAGGCGAAGAGGATGCCGAG     780
LDBPK_352470.1    GCCGTGAGCAACTTCGCCGCCTCTTTTGGGCTGCTTGCGGAAGGCGAAGAGGATGCCGAG     780
                  ************************************************************

LdSNXi            CTGCGCGGCGCCATCGAAGGGGTGCGCGACTGCGGTCGCAAAGTGGCAGATGTCTACAGC     840
LDBPK_352470.1    CTGCGCGGCGCCATCGAAGGGGTGCGCGACTGCGGTCGCAAAGTGGCAGATGTCTACAGC     840
                  ************************************************************

LdSNXi            AAACACGCTGACAACGAGTCGACGCGGCTTGTCAGCACGTTAAGCTTTTACGTGGGTATG     900
LDBPK_352470.1    AAACACGCTGACAACGAGTCGACGCGGCTTGTCAGCACGTTAAGCTTTTACGTGGGTATG     900
                  ************************************************************

LdSNXi            TGTGCCGCCGTGCGGGAGACTCTGAGCCACATGTTCAGCGCCCGCCAGTATCTGCGCAAC     960
LDBPK_352470.1    TGTGCCGCCGTGCGGGAGACTCTGAGCCACATGTTCAGCGCCCGCCAGTATCTGCGCAAC     960
                  ************************************************************

LdSNXi            TTGCAGAAGAAGGGCCAGGAACTGCAGGCGTCGGCGATGCGGGCACAGTCGGCCAACCAG     1020
LDBPK_352470.1    TTGCAGAAGAAGGGCCAGGAACTGCAGGCGTCGGCGATGCGGGCACAGTCGGCCAACCAG     1020
                  ************************************************************

LdSNXi            GTGCAGCTGCAGAGCGAGCTGCACTTCGTGAATGAGCAGCGGGCCCACCTCGAGGAAGAC     1080
LDBPK_352470.1    GTGCAGCTGCAGAGCGAGCTGCACTTCGTGAATGAGCAGCGGGCCCACCTCGAGGAAGAC     1080
                  ************************************************************

LdSNXi            CTCATGGGCGCCGAGAAGACCTTCAGCGAGGAATTTATTCTCTTCCACGAAAACAAGCAA     1140
LDBPK_352470.1    CTCATGGGCGCCGAGAAGACCTTCAGCGAGGAATTTATTCTCTTCCACGAAAACAAGCAA     1140
                  ************************************************************

LdSNXi            TATGACGCCAAGGATATGCTGAAGAAGTTTGGCATGCTGGAGCTCAGCTTCTCTGAGTCG     1200
LDBPK_352470.1    TATGACGCCAAGGATATGCTGAAGAAGTTTGGCATGCTGGAGCTCAGCTTCTCTGAGTCG     1200
                  ************************************************************

LdSNXi            ATGAAGCAGGAGTGGGACGCCCTGCGGCCAATGTTGGAGTCCCTTGGCACATAG           1254
LDBPK_352470.1    ATGAAGCAGGAGTGGGACGCCCTGCGGCCAATGTTGGAGTCCCTTGGCACATAG           1254
                  ******************************************************
```

CLUSTAL O(1.2.4) multiple sequence alignment
Amino acid Sequence

```
LdSNXi              MAAGNSISVKVDVPSQVKGQGTLEMSYYIYPITMRLPGFMSDARFNRRYTDFETLRGQLC       60
LDBPK_352470.1      MAAGNSISVKVDVPSQVKGQGTLEMSYYIYPITMRLPGFMSDARFNRRYTDFETLRGQLC       60
                    ************************************************************

LdSNXi              ATYWYCIVPPIPEKESVQDKLGKLPRMVASAKETTASEGDLLEYRRISLRRFLQRLAYHP       120
LDBPK_352470.1      ATYWYCIVPPIPEKESVQDKLGKLPRMVASAKETTASEGDLLEYRRISLRRFLQRLAYHP       120
                    ************************************************************

LdSNXi              ILGKSDLLQKFTNDNEWRQCTRDPVKPPRFIVSSSLEEIARSWAPSSSASGAGTQGSGGA       180
LDBPK_352470.1      ILGKSDLLQKFTNDNEWRQCTRDPVKPPRFIVSSSLEEIARSWAPSSSASGAGTQGSGGA       180
                    ************************************************************

LdSNXi              AGGGGVSQTGAAYQLALTQEPVDEATWRATSEYIGELESNLKSMRNLLEALVDRHRRAAS       240
LDBPK_352470.1      AGGGGVSQTGAAYQLALTQEPVDEATWRATSEYIGELESNLKSMRNLLEALVDRHRRAAS       240
                    ************************************************************

LdSNXi              AVSNFAASFGLLAEGEEDAELRGAIEGVRDCGRKVADVYSKHADNESTRLVSTLSFYVGM       300
LDBPK_352470.1      AVSNFAASFGLLAEGEEDAELRGAIEGVRDCGRKVADVYSKHADNESTRLVSTLSFYVGM       300
                    ************************************************************

LdSNXi              CAAVRETLSHMFSARQYLRNLQKKGQELQASAMRAQSANQVQLQSELHFVNEQRAHLEED       360
LDBPK_352470.1      CAAVRETLSHMFSARQYLRNLQKKGQELQASAMRAQSANQVQLQSELHFVNEQRAHLEED       360
                    ************************************************************

LdSNXi              LMGAEKTFSEEFILFHENKQYDAKDMLKKFGMLELSFSESMKQEWDALRPMLESLGT          417
LDBPK_352470.1      LMGAEKTFSEEFILFHENKQYDAKDMLKKFGMLELSFSESMKQEWDALRPMLESLGT          417
                    *********************************************************
```
