# Supplementary material for: Characterization of the First Secreted Sorting Nexin Identified in the Leishmania Protists
Source: Int J Mol Sci. 2024 Apr 7;25(7):4095. doi: 10.3390/ijms25074095 (PMC11012638; doi:10.3390/ijms25074095)
Supplement: Supplementary file 1 [file ijms-25-04095-s001.zip › SUPPLEMENTARY MATERIAL.pdf]

SUPPLEMENTARY MATERIAL

TABLES

Supplementary Table S1

Primer sequences used in the *Ld*SNXi cloning

No	Name of primer	Sequence
Primer 1	5’ BglII LDBPK.352470	5’-GAAGATCTACCATGGCCGCCGGCAACTCC-3’
Primer 2	3’ BamHI LDBPK.352470	5’-CGGGATCCAGGTCCTGTGCCAAGGGACTCCAAC-3’
Primer 3	5’ EcoRI LDBPK.352470	5’-CCGGAATTCATGGCCGCCGGCAACTCC-3’
Primer 4	3’ EcoRI LDBPK.352470	5’-CCGGAATTCCTATGTGCCAAGGGACTCCAAC-3’
Primer 5	5’ EcoRI LDBPK.352470_AA	5’-CCGGAATTCGAAGGGGTGCGCGACTG-3’

FIGURE LEGENDS

Supplementary Figure S1

Secondary structure prediction of *Ld*SNXi. Secondary structure prediction using the PsiPred algorithm. Amino acids forming the PX and BAR domains are bracketed by black-colored dotted lines. Residues predicted to be found in β-sheets are shown in yellow-colored boxes, while a-helices are shown in pink-colored boxes. As input for the modeling was used the entire sequence of *Ld*SNXi was used.

Supplementary Figure S2

Schematic representation of the protein products encoded by the plasmids used in this work. The name of each plasmid, the way it was used in this study and the associated results are mentioned alongside each construct representation. The total number of amino acids and the calculated molecular weights of the recombinant protein encoded by each construct are shown on the right. The start and end residue numbers of the *Ld*SNXi protein sequences included in each recombinant hybrid *Ld*SNXi forms are indicated at the top of each construct’s schematic diagram. A, B, C, D are indicating the different constructs.

Supplementary Figure S3

Purification stages of GST-*Ld*SNXi and GST produced in bacteria. Bacteria harboring the pGEX4T1-*ldsnxi* plasmid (**A**) or pGEX4T1 plasmid (**B**) were used for the induction of the corresponding protein. After lysis, the soluble fraction was used for incubating GST-Agarose beads and the bound protein was eluted as described in Materials and Methods. Total bacteria lysates before and after the induction, insoluble fraction, soluble fraction before and after binding, and eluted fractions were analyzed by SDS-PAGE [10% (w/v), 1.5 mm gel thickness]. The gel was stained with Coomasie. The band corresponding to the protein expressed and purified in each occasion is indicated with an arrow. Molecular weights are indicated in kDa.

Supplementary Figure S4

Generation and verification of specific a-*Ld*SNXi polyclonal antibody

A. Coomasie Stained SDS PAGE gel of bacteria lysates' soluble and insoluble fractions examining the expression of MBP-*Ld*SNXi-C-term in different IPTG concentrations. **B**. Purification steps of MBP-*Ld*SNXi-C-term used as antigen in the rabbit immunization. **C**. SDS PAGE gel on which were analyzed soluble and insoluble fractions of bacteria lysates for the expression of GST-*Ld*SNXi-C-term in different conditions. **D.** Western Blot analysis of the a-*Ld* SNXi pAbs' reactivity (rabbit polyclonal) in detecting the GST-*Ld*SNXi-C-term protein produced in *E. coli* BL21 cells carrying the *pGEX-4T1-ldsnxi-c-term* plasmid.

Supplementary Figure S5

Biochemical detection of the endogenous *Ld*SNXi in *wt L. donovani* (LG13) promastigotes. Total lysates (60 μg protein/well) from logarithmic (log) phase cultures of promastigotes were analyzed by SDS-PAGE [10% (w/v), 1.5 mm gel thickness] and immunoblotted with the purified rabbit a-SNXi pAb (0.5 μg/ml) (**a**). The membrane stained with Ponceau-S is shown (**b**) as a loading indicator. Molecular weights are indicated in kDa. The arrow on the right indicates the migration position of *Ld*SNXi.

Supplementary Figure S6

Schematic representation of Blastp pairwise sequence alignment of *Ld*SNXi (*Ld*BK 352470.1 gene product) and human PX-BAR sorting nexins. (A) depicts only the statistically significant matches calculated by the algorithm and the query coverage of the alignment of PX domains while **(B)** depicts only the statistically significant matches calculated by the algorithm and the query coverage of the alignment of BAR domains.

Supplementary Figure S7

Evolutionary structural conservation of the *Ld*SNXi PX and BAR domains.

Structural alignment of the 3D structures, of **(A)** the PX domains and **(B)** the BAR domains of human SNX1 and *Ld*SNXi protein, respectively. The solved structures of PX and BAR domains of SNX1 were obtained from the Protein Data Bank (PDB) under accessions 2I4K and 4FZS. The predicted structure of *Ld*SNXi was retrieved from AlphaFold. The comparative analysis is visualized with PyMol. The PX domain of *Ld*SNXi protein is colored in red, the BAR domain in blue, and the coil in yellow. The PX domain of the human SNX1 is colored in pink, and the BAR domain in light blue/grey.

Supplementary Figure S8

Cell cycle dependent localization of heterologously expressed r*Ld*SNXi-EGFP in HeLa cells. Different cell cycle dependent morphological forms of transfected HeLa cells stained without pre-extraction for α-tubulin and F-Actin by IF with the mouse anti-α-Tubulin pAb (1: 2,000) followed by secondary Abs conjugated to CF®546 and Phalloidin-CF 633 (1: 1,000). Nuclear DNA was stained with Hoechst 33342 (1:5,000). Representative images of different cell cycle morphological forms were acquired by z-scanning performed at 0.5 μm step size using the TCS SP8 Leica confocal microscope. A single z section with representative staining from each case is shown. Single FL images are shown in black and white (BW) for better contrast while images of the merged FL signals are shown in color. The molecule highlighted in the BW images with single color FL is indicated at the top in the same color as the respective FL signal. Scale bar size: 5 μm. **A**. Interphase cells. **B**. A cell in prometaphase. **C**. A cell in anaphase. **D**. A cell in anaphase. **E**. Cells in early telophase. **F**. Cells in late telophase. Circles in **A** highlight the co localization of *Ld*SNXi-EGFP signal to that of microtubules. Circles in **B** highlight the accumulation of *Ld*SNXi-EGFP around chromosomes. Arrows in **C** point to one of the poles of the mitotic spindle, in **E** the cleavage furrow and in **F** the intercellular bridge.

Supplementary Figure S9

Protein-Protein Interaction (PPI) networks of human SNX1, SNX2, SNX4 and SNX7 as predicted by STRINGdb. The line thickness indicates the strength of data support. **A**. Network for SNX1 interacting partners. **B**. Network for SNX2 interacting partners. **C**. Network for SNX4 interacting partners. **D**. Network for SNX7 interacting partners
