# Supplementary figures and images for: Characterization of the First Secreted Sorting Nexin Identified in the Leishmania Protists
Source: Int J Mol Sci. 2024 Apr 7;25(7):4095. doi: 10.3390/ijms25074095 (PMC11012638; doi:10.3390/ijms25074095)

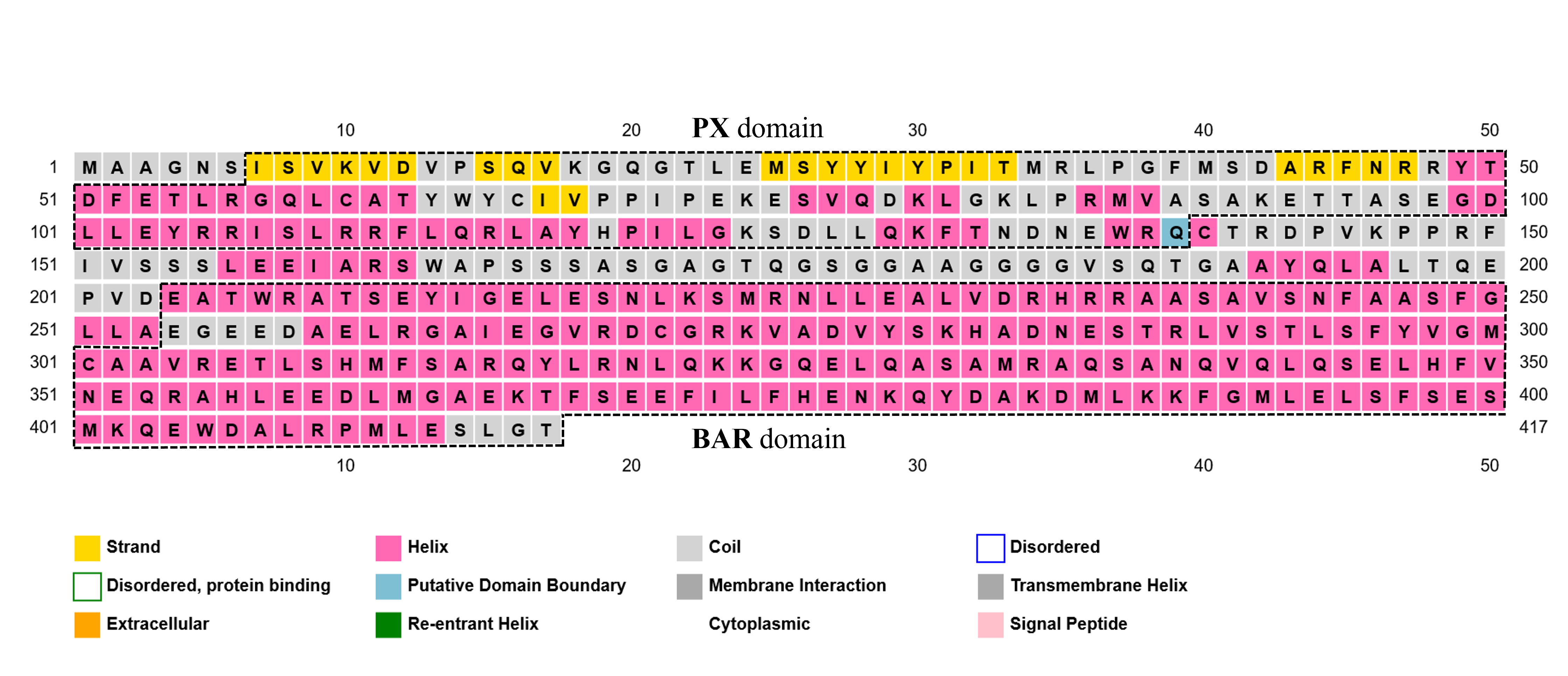

Supplement: Supplementary file 1 [file ijms-25-04095-s001.zip › Supplementary Figure S1.png]

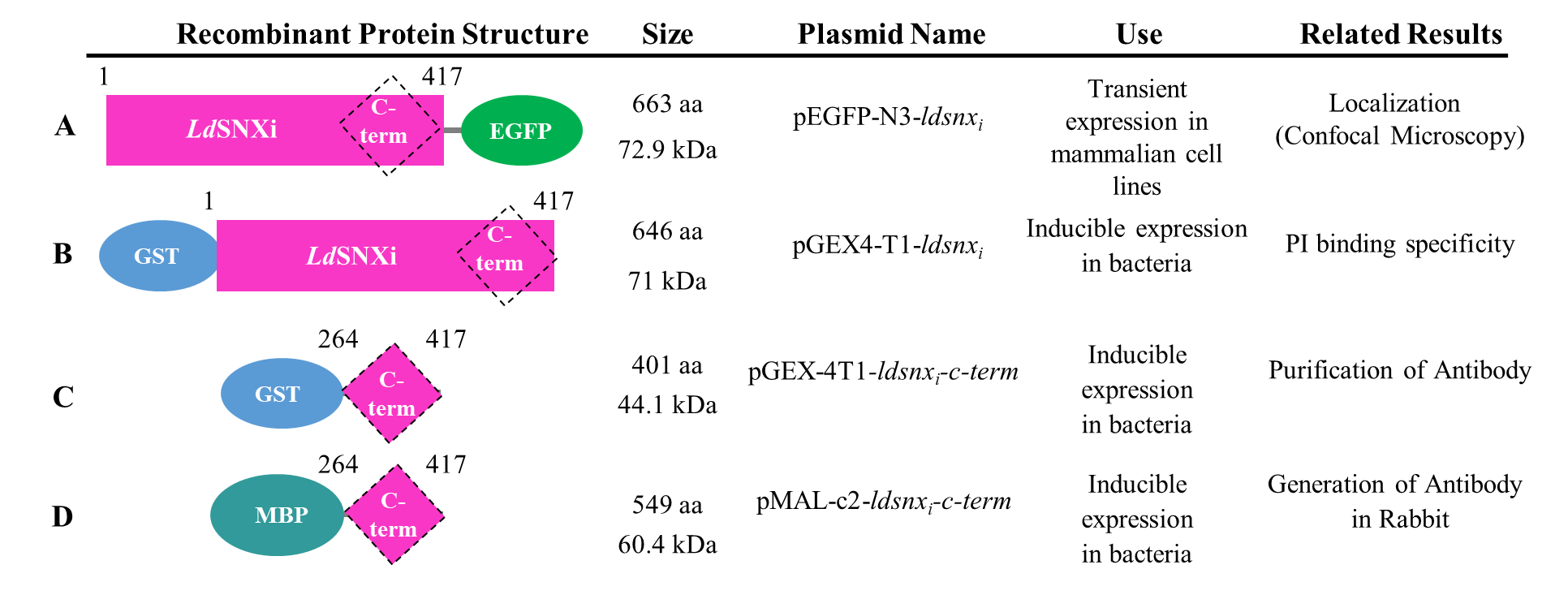

Supplement: Supplementary file 1 [file ijms-25-04095-s001.zip › Supplementary Figure S2.png]

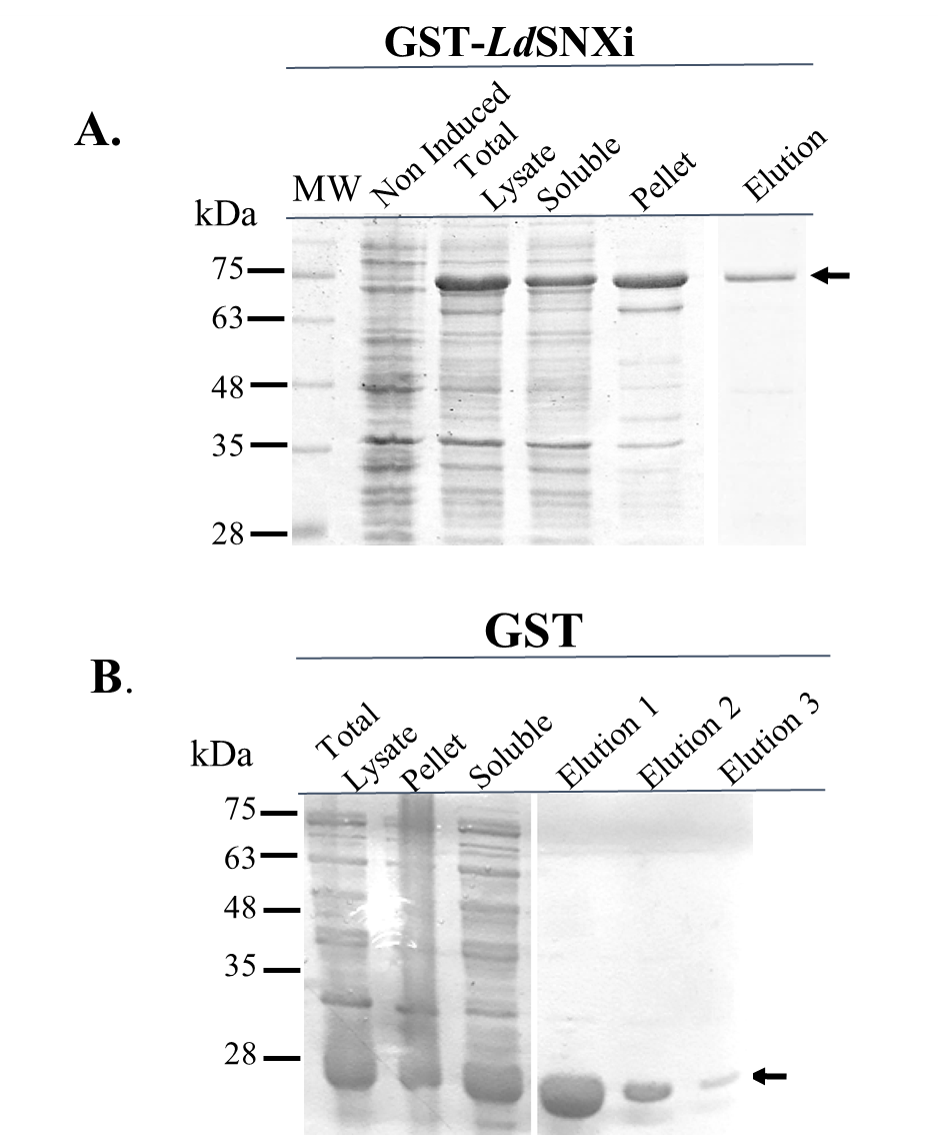

Supplement: Supplementary file 1 [file ijms-25-04095-s001.zip › Supplementary Figure S3.png]

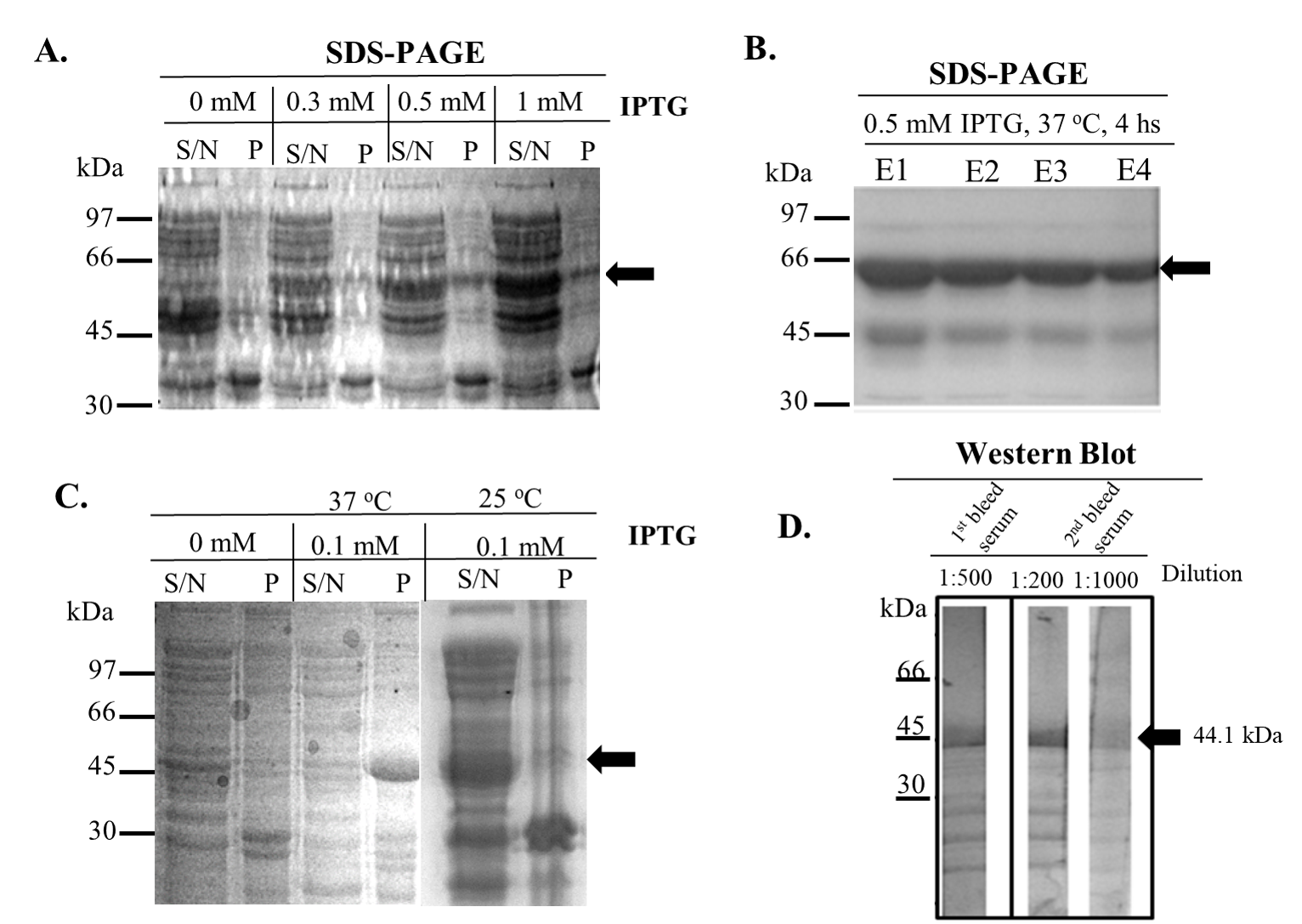

Supplement: Supplementary file 1 [file ijms-25-04095-s001.zip › Supplementary Figure S4.png]

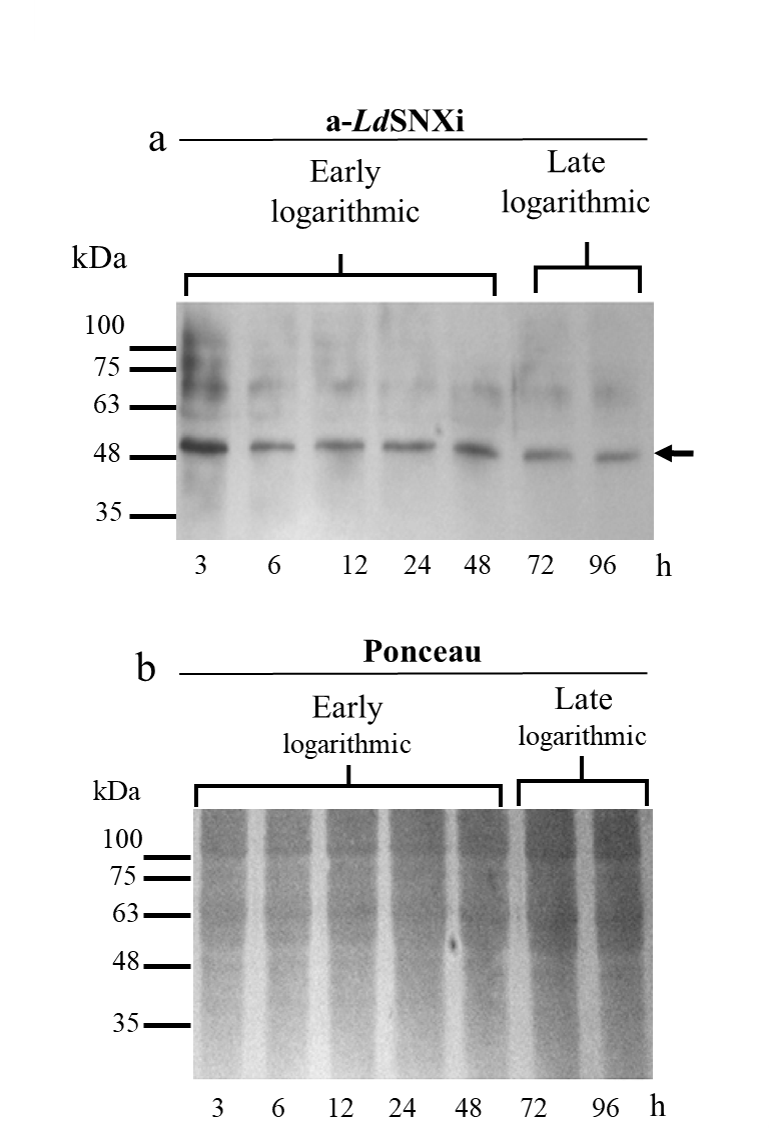

Supplement: Supplementary file 1 [file ijms-25-04095-s001.zip › Supplementary Figure S5.png]

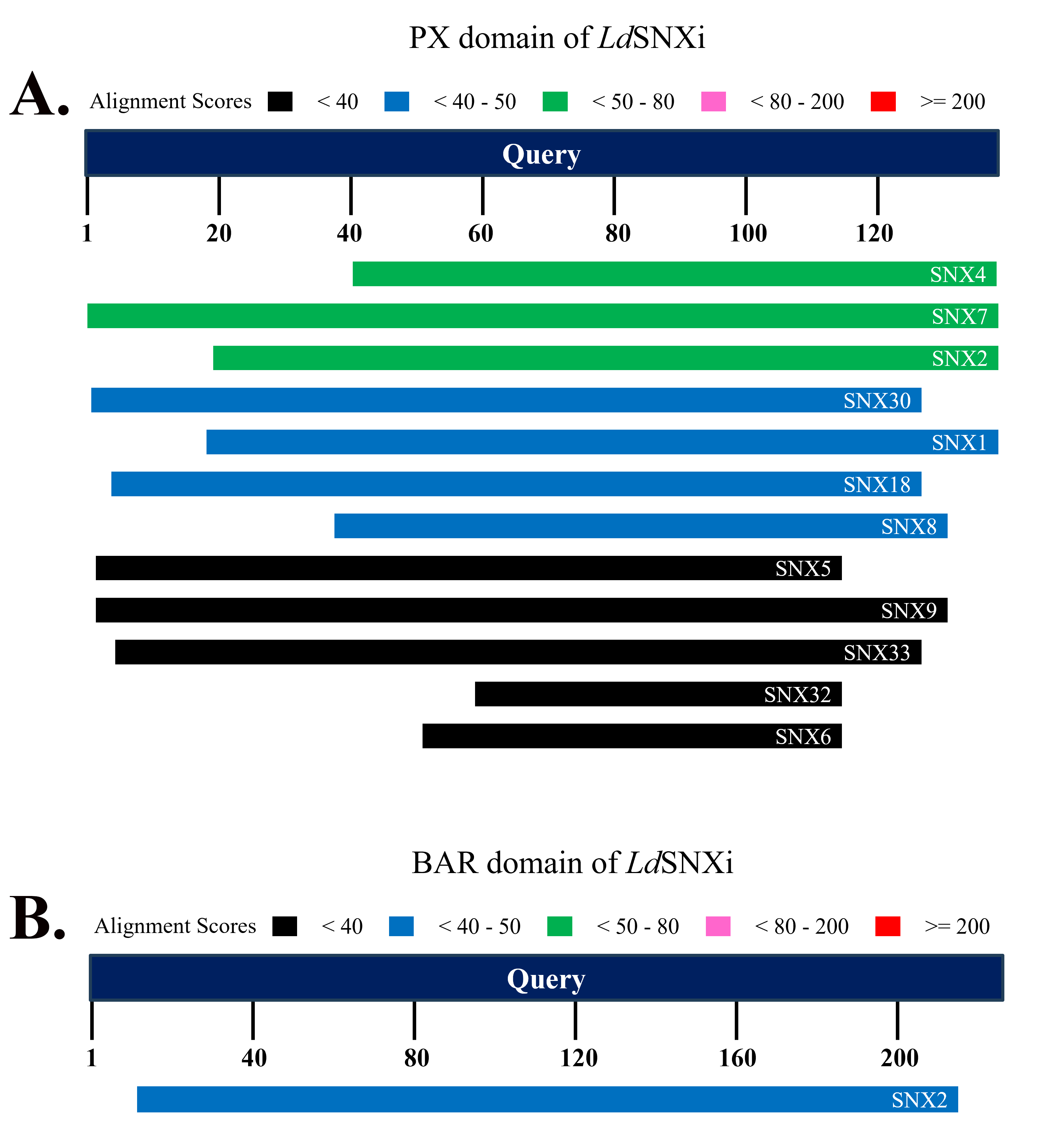

Supplement: Supplementary file 1 [file ijms-25-04095-s001.zip › Supplementary Figure S6.png]

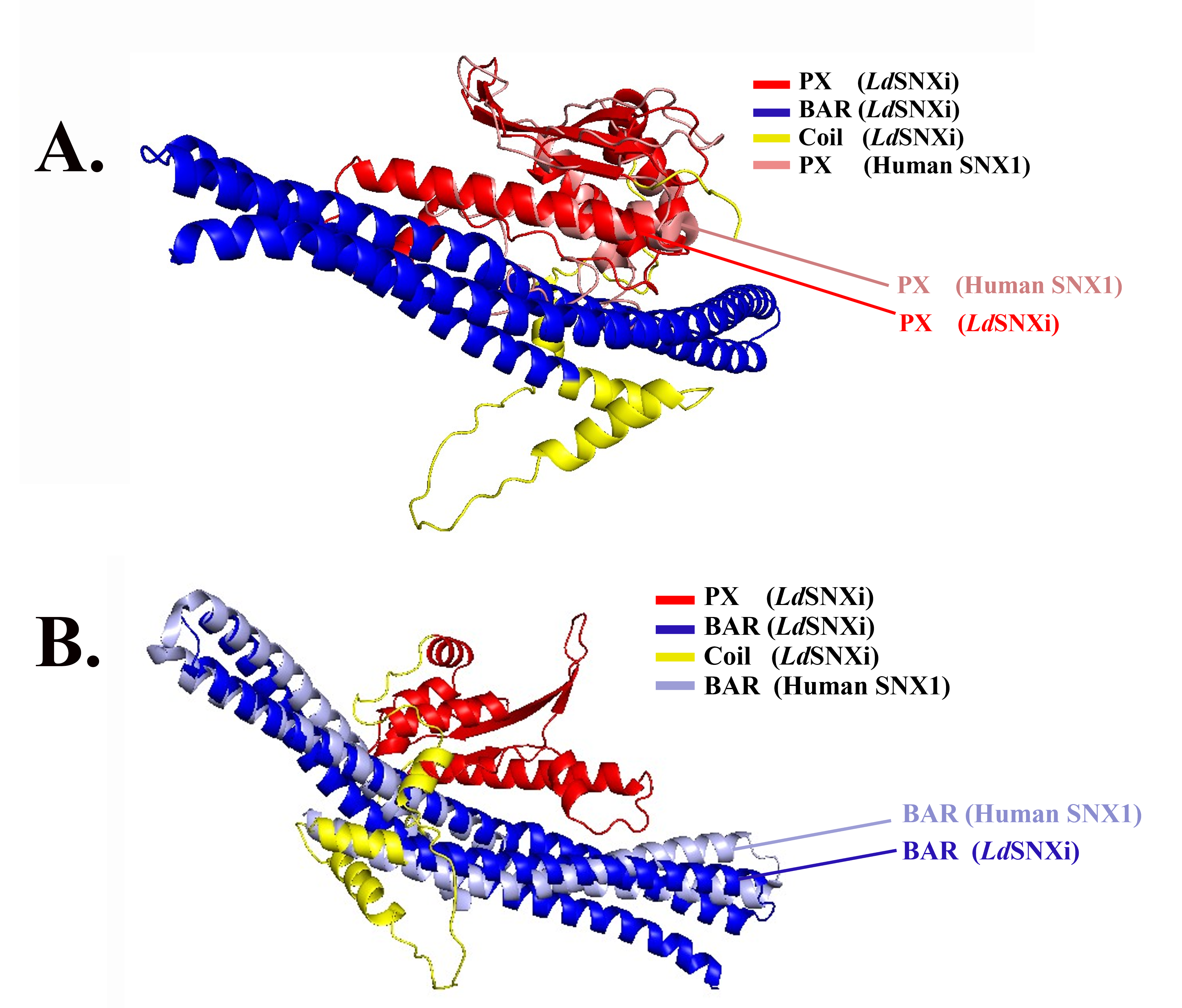

Supplement: Supplementary file 1 [file ijms-25-04095-s001.zip › Supplementary Figure S7.png]

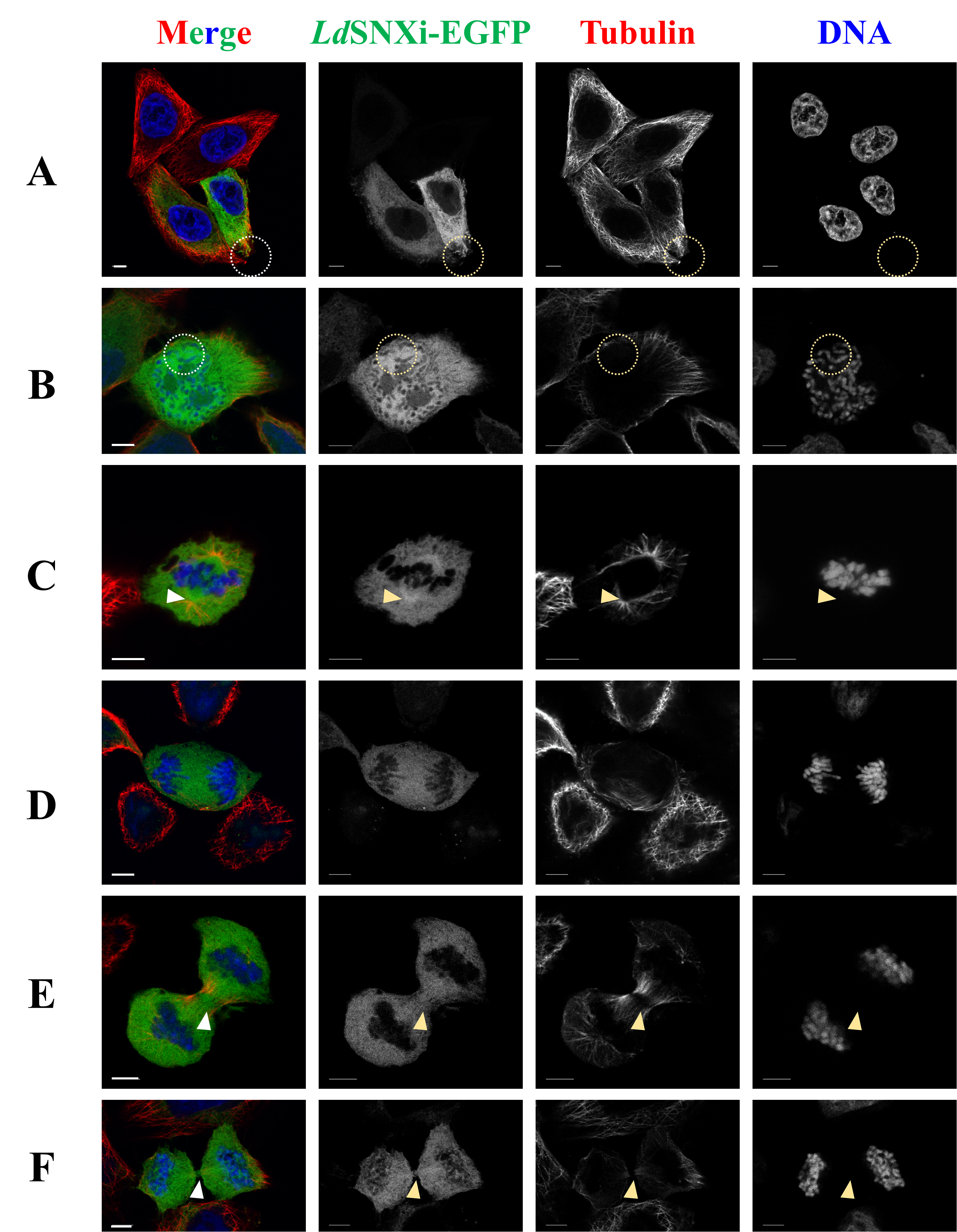

Supplement: Supplementary file 1 [file ijms-25-04095-s001.zip › Supplementary Figure S8.png]

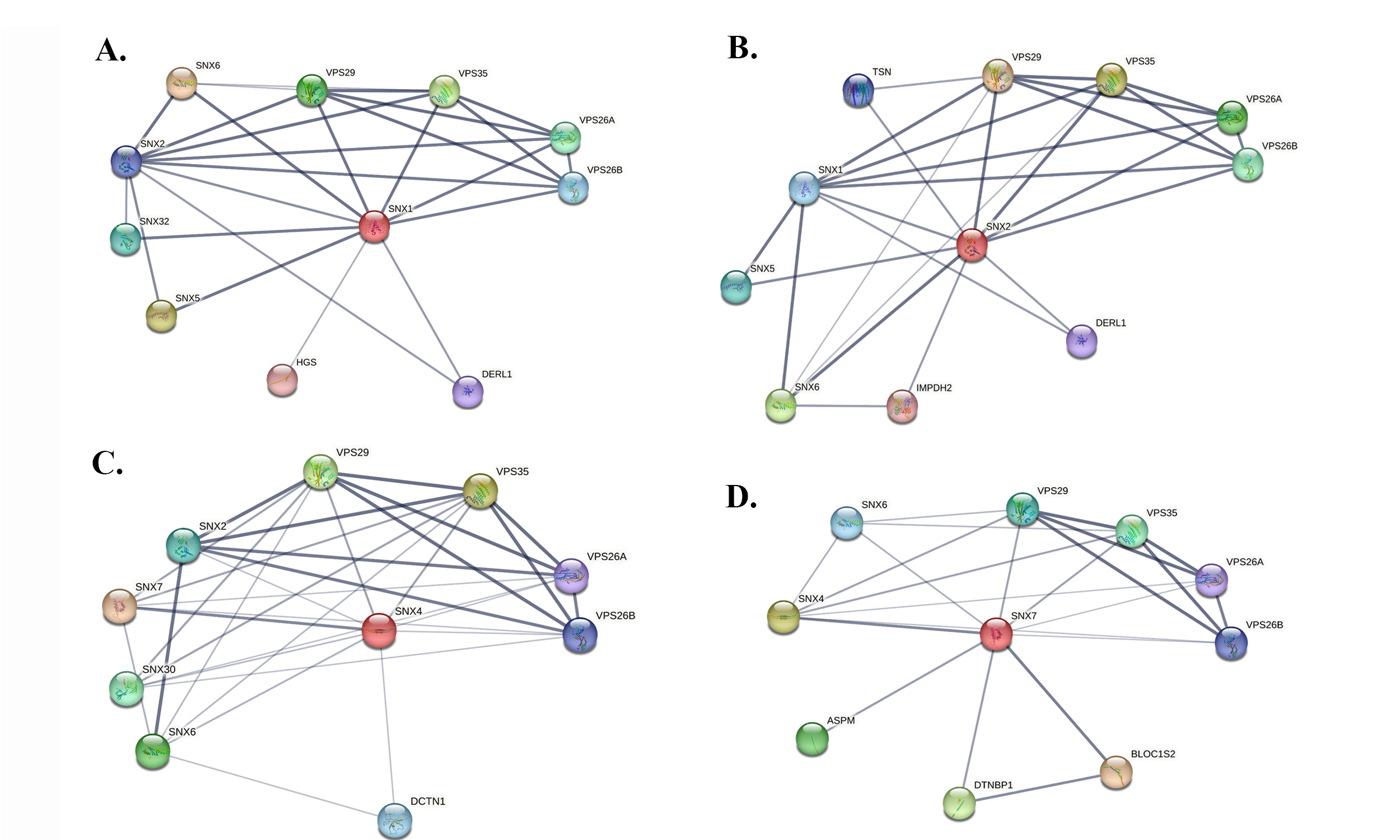

Supplement: Supplementary file 1 [file ijms-25-04095-s001.zip › Supplemetary Figure S9.png]
